# The effect of number of healthcare visits on study sample selection in electronic health record data

**DOI:** 10.23889/ijpds.v5i1.1156

**Published:** 2020-04-02

**Authors:** LJ Rasmussen-Torvik, A Furmanchuk, AJ Stoddard, AI Osinski, JR Meurer, N Smith, E Chrischilles, BS Black, A Kho

**Affiliations:** 1 Department of Preventive Medicine, Northwestern University Feinberg School of Medicine, Chicago, IL 60611; 2 Center for Health Information Partnerships, Northwestern University Feinberg School of Medicine, Chicago, IL 60611; 3 Clinical and Translational Science Institute/Institute for Health & Equity, Medical College of Wisconsin, Milwaukee, WI, 53226; 4 Department of Epidemiology, College of Public Health, University of Iowa, Iowa City, 52242; 5 Pritzker School of Law and Kellogg School of Management, Northwestern University, Chicago, IL 60611

## Abstract

**Introduction:**

Few studies have addressed how to select a study sample when using electronic health record (EHR) data.

**Objective:**

To examine how changing criterion for number of visits in EHR data required for inclusion in a study sample would impact one basic epidemiologic measure: estimates of disease period prevalence.

**Methods:**

Year 2016 EHR data from three Midwestern health systems (Northwestern Medicine in Illinois, University of Iowa Health Care, and Froedtert & the Medical College of Wisconsin, all regional tertiary health care systems including hospitals and clinics) was used to examine how alternate definitions of the study sample, based on number of healthcare visits in one year, affected measures of disease period prevalence. In 2016, each of these health systems saw between 160,000 and 420,000 unique patients. Curated collections of ICD-9, ICD-10, and SNOMED codes (from CMS-approved electronic clinical quality measures) were used to define three diseases: acute myocardial infarction, asthma, and diabetic nephropathy).

**Results:**

Across all health systems, increasing the minimum required number of visits to be included in the study sample monotonically increased crude period prevalence estimates. The rate at which prevalence estimates increased with number of visits varied across sites and across diseases.

**Conclusion:**

In addition to providing thorough descriptions of case definitions, when using EHR data authors must carefully describe how a study sample is identified and report data for a range of sample definitions, including minimum number of visits, so that others can assess the sensitivity of reported results to sample definition in EHR data.

**Key words:**

Electronic Health Records, Sampling Studies, Prevalence, Methods

## Introduction

Increased adoption of electronic health records (EHR) has generated increased interest in using these data in clinical and epidemiologic research [1]. EHRs have been proposed as offering an efficient means for identifying eligible subjects for retrospective studies and for prospective observational studies or pragmatic trials. EHRs can offer extensive data elements that are desirable for capturing baseline inclusion and exclusion criteria, covariates, treatments and interventions, and study outcomes.

EHRs can provide a reasonably complete picture of a patient’s health and medical encounters in “closed” health systems, such as traditional health maintenance organizations. However, many EHRs are drawn from non-closed systems, which likely provide only some of a patient’s health care encounters. The EHR will often reflect a subset (sometimes a small subset) of patient encounters and as a result, diagnoses. Therefore, it is challenging to define a study sample using EHR data from a non-closed system, and, therefore, to calculate even basic outcomes such as chronic disease prevalence. Researchers must specify criteria for determining which patients have sufficient information in the EHR to be included in the study sample. Often, this sample is defined by requiring that persons have a minimum number of visits in a defined period (for example two visits in a three year period) [2].

Many studies have been published that propose, validate, and, in some cases, compare disease definitions in EHR systems (see Sprat et al. [3] for a type 2 diabetes example and Pathak et al. for an overview) [4]. One study included a simulation demonstrating that, for lower-sensitivity phenotypes, the potential for bias is exacerbated when the medical condition also leads to more patient encounters [5]. However, the specific implications of different methods for selecting a study sample have received little examination. The objective of this report was to examine how changing criterion for the minimum number of visits in EHR data required for inclusion in a study sample would impact one basic epidemiologic measure: estimates of disease period prevalence, i.e. the proportion of individuals in a defined population that have a disease during a specified time period. Period prevalence of three common diseases was examined across three large geographically and demographically diverse health care systems. This article demonstrates a critical issue that must be addressed before EHR records from non-closed systems can routinely be used in studies involving prevalence in population data science studies [6].

## Methods

EHR data from three different health systems participating in the Greater Plains Collaborative [7] and Chicago-Area (CAPriCORN) [8] Clinical Research Networks (CRNs) was used. Systems with varying locations and diverse population demographic populations served were intentionally chosen. University of Iowa Health Care consists of one academic medical center that includes 32 adult and 13 pediatric outpatient primary care clinics. In addition, there are 47 adult and 11 pediatric specialty or surgical clinics. They serve patients from eight states. Froedtert & the Medical College of Wisconsin is an integrated health care system that provides health-related services including hospitals and health centers, home care, laboratory, health insurance, employer health services and workplace clinics, and digital health solutions. Froedtert combines with MCW to form eastern Wisconsin’s only academic medical center and associated regional health network supporting a shared mission of patient care, innovation, medical research and education. Froedtert provides more than 1,000 beds and with MCW includes 1,700 physicians, 4,100 nurses, and 15,000 other staff. Northwestern Memorial Health Care has over 4000 affiliated physicians and 30,000 employees who see patients at over 200 hospital and clinic sites. In 2016, Northwestern Medicine hospitals included Northwestern Memorial Hospital, a large, urban, academic, teaching hospital and Level One Trauma Center, with 894 beds in downtown Chicago, and Northwestern Medicine Lake Forest Hospital, a 118-bed community hospital located about 30 miles north of downtown Chicago.

Each health system provided inpatient, outpatient and emergency department diagnoses (using ICD9, ICD10 and SNOMED codes) for all patients over the age of 18 with health care encounters on two or more discrete days during 2016. The initial sample was filtered to require at least two “visits” (defined as any Ambulatory Visit, Emergency Department Visit, Emergency Department Admit to Inpatient Hospital Stay, Inpatient Hospital Stay, Non-Acute Institutional Stay, Observation Stay, Institutional Professional Consult, or Other Ambulatory visit) per the People-Centered Outcomes Research Institute Common Data Model (https://pcornet.org/pcornet-common-data-model/), and varied the minimum number of visits from two to six.

To define cases of specified diseases in the study samples, curated collections of ICD-9, ICD-10, and SNOMED codes drawn from the Center for Medicare Studies Electronic Clinical Quality Measures (eCQMs—https://ecqi.healthit.gov/ecqms) were used. For myocardial infarction, codes for the denominator of the eQCM “Coronary Artery Disease: Beta-Blocker Therapy-Prior Myocardial Infarction (MI) or Left Ventricular Systolic Dysfunction (LVEF <40%)” [9] were used; for diabetic nephropathy codes for the numerator of the eQCM “Diabetes: Medical Attention for Nephropathy” [10] were used; and for persistent asthma codes for the denominator of the eQCM “Use of appropriate medications for asthma” [11] were used. An individual was treated as having one of these three conditions in 2016 if the EHR indicated a disease specific code on any date in 2016. Data were analyzed in 2018. We chose these three conditions as they were important causes of morbidity in the US, diverse in their clinical presentation (acute vs. chronic), and had variable average ages of onset. 

### Statistical analysis

Crude period prevalence (prevalent case count / study sample) during 2016 was calculated for each disease and each network, based on different study sample definitions that required two, three, four, five or six visits to the health care system between January 1 2016 and December 31 2016). This analysis was also repeated restricting the study population to patients between the ages of 46 and 65 in 2016.

## Results

Table 1 presents the demographics of patients seen at least two times at each of the three hospital and clinic systems in 2016. The number of patients seen at least two times (i.e. the study sample that required two visits) ranged from 131,000 to 345,000 across the sites. At all three sites around 40% of the patients were male, and the mean age was between 40 and 45 at all sites. The majority of patients were white at all three sites. Although all three health systems included large hospitals, less than 2.5% of visits at each site were coded as inpatient visits.

Figure 1 shows how the study sample for each health system changed as the required number of visits to that health system during 2016 increased. In all cases, not surprisingly, increasing the minimum number of visits required dramatically reduced the size of the sample. Interestingly, the sites showed similar relative rates of decline in the sample sizes as the number of required visits increased. For all three sites, requiring six visits nearly halved the sample size, compared to requiring only two visits.

Figure 2 shows how the calculations of crude period prevalence (2016) for myocardial infarction (MI) (a) diabetic nephropathy (b) and persistent asthma (c) changed across health systems, as the minimum number of health system visits required for inclusion in the study sample increased. The number of patients with each of these diagnoses also fell as the minimum number of visits increased, but much more slowly than the denominator. As a result, the prevalence of all three diseases increased as the number of visits required to enter the study sample increased across all health systems. However, the rate of increase differed across sites and diseases. For site 2 increasing the number of required visits from two to six increased MI prevalence by 36% and increased persistent asthma prevalence by 48%. For MI, increasing the number of required visits from two to six increased MI prevalence at site 1 by 57% and MI prevalence at site 2 by 36%. To see if some standardization of populations across the three sites reduced observed differences in the way prevalence changed with increasing number of required visits, we also calculated crude period prevalence (2016) for MI, diabetic nephropathy, and persistent asthma by the minimum number of health system visits required for inclusion in the subset of 46-65 year-olds (Table 2). This attempt at standardization actually increased observed differences in the way MI prevalence changed with increasing number of required visits across sites; increasing the number of required visits from two to six in the subset of 46-65 increased MI prevalence at site 1 by 68% and MI prevalence at site 2 by 37%.

## Discussion

This study demonstrates that for three disease conditions, across three different health systems, estimates of basic descriptive epidemiology metrics, including crude disease period prevalence, change as one increases the minimum number of health care system visits required to be included in the study sample (without any change in the case definition). The increase in prevalence with minimum number of visits is not surprising in two respects. First, people with more contact with the health care system will have more complete records of their health status. Secondly as one increases the number of visits required to enter a study sample, one loses healthy individuals (who presumably have less contact with any health care system), making results less generalizable to the population. Unfortunately, there appears to be only limited consistency in the magnitude of increases in period prevalence estimates as one increases the minimum number of required visits across different diseases (within a single health care system) and across health care systems (for a single disease). This limits generalizable recommendations about study sample definition using EHRs from non-closed systems. Given these challenges (as well as lack of adjustment, different measurement methods, and geographic and demographic diversity) it is not surprising that the calculated crude estimates of MI, diabetic nephropathy, and asthma differed both widely across health systems and from recent Behavioral Risk Factor Surveillance System and National Health Interview Survey estimates [12-14]. Our study presents only results from three sites, and, almost certainly, specific results would have been different with the use of different clinical sites. However, the inconsistency in the magnitude of increases in period prevalence across sites and across diseases with increasing number of visits required to enter the study sample shows the challenges in establishing standard rules or practices about how to define a study sample in EHR data. There would surely be further variation if one compared health systems such as the tree we study, which include extensive clinic networks, with narrower systems.

The complexity of defining a *study sample* in EHR data has been largely ignored in the biomedical literature. However, previous studies have examined the role number of visits should potentially play in *case definitions* from EHR data. In a validation study of an EHR algorithm for rheumatoid arthritis using only ICD-9 codes, increasing the number of distinct ICD-9 codes required for a case definition increased the positive predictive value across three different sites from 33 to 57 percent [15]. Diabetes DataLink required repeated outpatient visits in order to classify someone as a case of prevalent diabetes (but not repeated inpatient visits); “prevalent diabetes cases were patients who met at least one of the following criteria within a 18-month period: 1+ diabetes-specific medication, 1+ inpatient diabetes diagnosis, 2+ face-to-face outpatient diabetes diagnoses on separate days, **or** 2+ elevated blood glucose values performed on separate days or one elevated oral glucose tolerance test” [16]. Because diagnosis codes can reflect only *possible* diagnoses when used to justify billing for screening tests, they may not always indicate the presence of a health condition, but rather the suspicion of a health condition. This could vary by care site. For example, in prior work, we identified that a diagnosis of chest pain was actually negatively correlated with the presence of a true myocardial infarction given how often a chest pain diagnosis code was used compared with patients later found to have an actual myocardial infarction [17]. Some EHR case definitions have even incorporated an element of number of visits over a very specific period of time; a published EHR case definition for heart failure defined patients having both an ICD-9 code and second mention of heart failure (extracted by NLP) within 365 days as having definite heart failure while a patient having both an ICD-9 code and second mention of heart failure (extracted by NLP) within 365-1825 days was classified only as having probable heart failure [18]. There is thus a growing (but still limited) understanding that repeated elements required for EHR case definitions can vary by disease, type of visit, and perhaps even by site. But there is not yet recognition that similar complexities may likely exist for the use of repeated elements in defining a study population. It is critical that these complexities begin to be recognized as more and more projects begin to use the EHR to estimate disease prevalence [19-21].

## Conclusion

This study provides evidence that decisions in the basic definition of the study population can greatly impact estimation of disease prevalence, which, in turn, can greatly impact many descriptive and analytic epidemiology analyses. Study population definition should be a carefully considered element in any clinical or epidemiologic study using EHR data. Investigators should also consider reporting the effect of different EHR study sample definitions on outcomes, and be aware that different definitions may be preferred for study of different diseases. If an algorithm for EHR study sample selection for a disease is proposed, it should be tested across multiple health systems, in the same manner that EHR phenotype definitions are often tested [22].

## Ethics statement

This study, “Natural Experiments in Diabetes Translation (NEXT-D)”, was reviewed and approved by the Chicago Area Institutional Review Board (CHAIRb FWA #00000083, IRB#0009693), Protocol #16030901.

Figures & Tables

**Table 1: Demographics of patients having at least two visits to each site in 2016:** 

	Site 1	Site 2	Site 3
N, patients seen at least twice in 2016 (to the nearest 1000)	345,000	131,000	198,000
Male, %	37.3	41.2	39.7
Mean age, years	44	42	45
Race, %

American Indian or Alaska Native	0.2	0.3	0.3
Asian	3.9	3.6	2.2
Black or African American	10.5	5.5	13.9
Native Hawaiian or Other Pacific Islander	0.1	0.1	0.1
White	62.5	84.3	79.1
Missing, not reported, or not captured in data model	22.8	6.2	4.4
Ethnicity %

Hispanic or Latino	7.7	3.2	4.0
Not Hispanic or Latino	77.6	95.3	95.9
Missing, not reported, or not captured in data model	14.7	1.5	0.1
Visits coded as inpatient %	1.4	1.1	2.4

**Figure 1: Study sample for each health system for different minimum required number of visits ([inpatient, outpatient, or emergency room visits] from 1st January 2016 to 31st December 2016):**
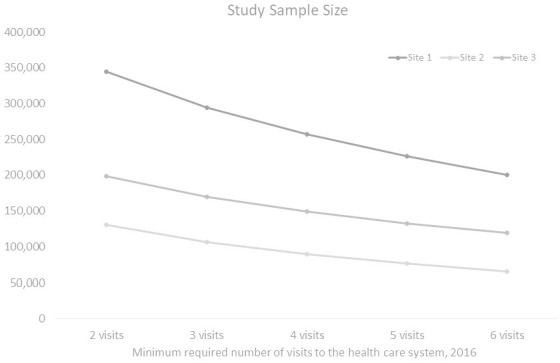


**Figure 2: Variation in basic descriptive epidemiology metrics (example: period prevalence) with increase in minimum number of health care visits required to be included in the study sample. Estimates of period prevalence during 2016 for (a) myocardial infarction (b) diabetic nephropathy and (c) persistent asthma, for different minimum numbers of required visits during 2016.**
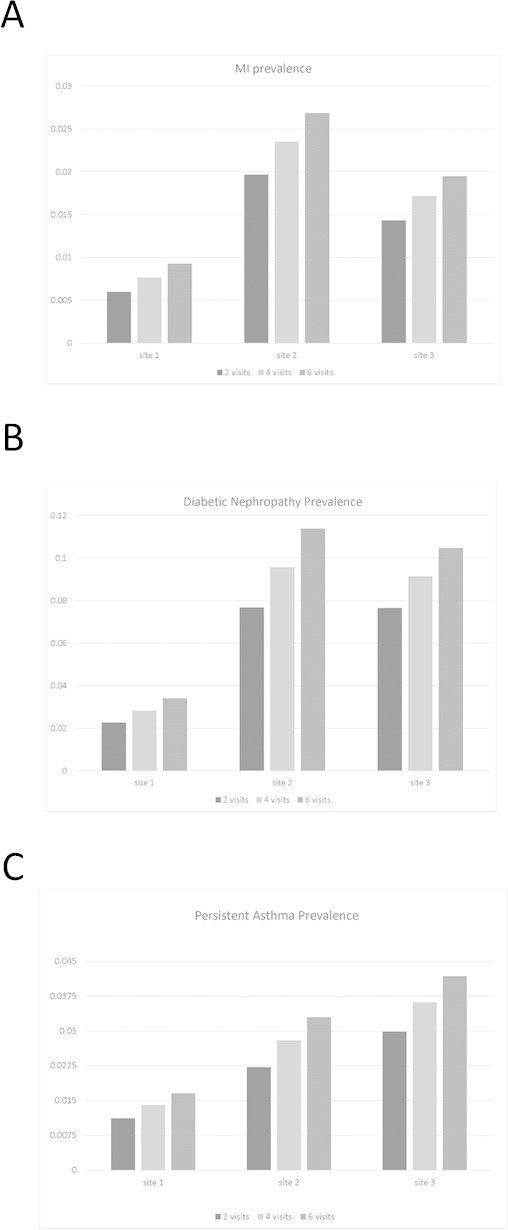


**Table 2: Variation in period prevalence (2016) of three diseases with increase in minimum number of health care visits required to enter the study sample, in participants aged 46-65, by site.** 

Disease	# of visits	Period Prevalence		
		Site 1	Site 2	Site 3
Myocardial Infarction	2 visits	0.0013	0.0030	0.0032
Myocardial Infarction	4 visits	0.0017	0.0040	0.0038
Myocardial Infarction	6 visits	0.0022	0.0048	0.0044
Diabetic Nephropathy	2 visits	0.0103	0.0320	0.0348
Diabetic Nephropathy	4 visits	0.0132	0.0415	0.0422
Diabetic Nephropathy	6 visits	0.0161	0.0510	0.0492
Persistent Asthma	2 visits	0.0098	0.0197	0.0234
Persistent Asthma	4 visits	0.0125	0.0248	0.0287
Persistent Asthma	6 visits	0.0148	0.0294	0.0335

## Abbreviations

Electronic Health Record	EHR
Clinical Data Research Network	CDRN
electronic Clinical Quality Metrics	eCQMs
Myocardial Infarction	MI
